# Rotational total skin electron irradiation with a linear accelerator

**DOI:** 10.1120/jacmp.v9i4.2793

**Published:** 2008-11-03

**Authors:** Eric P. Reynard, Michael D.C. Evans, Slobodan Devic, William Parker, Carolyn R. Freeman, David Roberge, Ervin B. Podgorsak

**Affiliations:** ^1^ Department of Medical Physics McGill University Health Centre Montreal Quebec Canada; ^2^ Department of Radiation Oncology McGill University Health Centre Montreal Quebec Canada

**Keywords:** total skin electron irradiation, rotational, treatment, linear accelerator

## Abstract

The rotational total skin electron irradiation (RTSEI) technique at our institution has undergone several developments over the past few years. Replacement of the formerly used linear accelerator has prompted many modifications to the previous technique. With the current technique, the patient is treated with a single large field while standing on a rotating platform, at a source‐to‐surface distance of 380 cm. The electron field is produced by a Varian 21EX linear accelerator using the commercially available 6 MeV high dose rate total skin electron mode, along with a custom‐built flattening filter. Ionization chambers, radiochromic film, and MOSFET (metal oxide semiconductor field effect transistor) detectors have been used to determine the dosimetric properties of this technique. Measurements investigating the stationary beam properties, the effects of full rotation, and the dose distributions to a humanoid phantom are reported. The current treatment technique and dose regimen are also described.

PACS numbers: 87.55.ne, 87.53.Hv, 87.53.Mr

## I. INTRODUCTION

We recently decommissioned a 30‐year‐old Varian Clinac 18 linear accelerator (Varian Medical Systems, Palo Alto, CA) which had been used clinically for routine external beam therapy and rotational total skin electron irradiation (RTSEI) treatments since the early 1980s.^(^ ^1^ ^–^ ^5^ ^)^ As a result, this RTSEI technique was transferred to a new Clinac 21EX linear accelerator (LINAC) equipped with an optional high‐dose total skin electron (HDTSe) mode that provides a 6 MeV electron beam with a dose rate of the order of 27 Gy/min at a source‐to‐skin distance (SSD) of 100 cm.^(6)^ ^,^ ^(7)^ By using a custom‐built flattening filter and delivering the treatment while the patient is standing on a rotating platform at a nominal SSD of 380 cm, we were able to reproduce our RTSEI technique that has been in clinical use since the early 1980s and to deliver the prescription target dose with a homogeneity in accordance with recommended guidelines.^(8)^

## II. METHODS

### A. Linear accelerator

**Figure 1:**
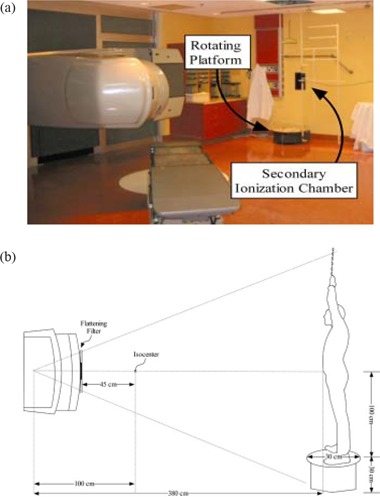
(a) Photograph of the treatment room showing the LINAC and couch positions, the rotating platform, the secondary ionization chamber, and the patient supports. (b) Diagram showing the room layout and treatment distances.

The 21EX LINAC is housed in a conventional radiation therapy treatment bunker. With the rotating platform located about 20 cm from the wall, the nominal treatment SSD is 380 cm. Fig. 1(a) is a photograph of the treatment room showing the LINAC and the rotating platform; Fig. 1(b) shows the geometry of the RTSEI.

Single‐beam RTSEI treatments require a very large field, of the order of 200 cm in height by 90 cm in width, to cover the entire patient surface. To achieve this field size, the RTSEI technique is delivered by placing the gantry laterally to 270 degrees and the collimator to 45 degrees (which maximize the vertical field and the horizontal field axis), and the couch to 45 degrees (to remove it from the beam's path). Upon insertion of the HDTSe accessory tray, the photon upper and lower jaws are custom configured to open to their largest available setting of 40 cm×40 cm to provide the largest possible electron field size. This 40 cm×40 cm collimator setting was configured using the “physics mode” on the console computer.

### B. Flattening filter

**Figure 2:**
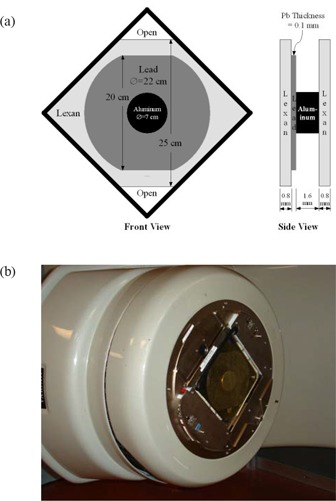
The custom‐built flattening filter constructed of aluminum, lead, and polymethyl methacrylate. (a) Schematic. (b) Photograph of the filter mounted on the linear accelerator accessory tray.

The 6 MeV electron beam used in our RTSEI technique lacks sufficient flatness to be used clinically, and therefore a custom‐built flattening filter is placed onto the LINAC's coded accessory tray. As shown schematically in Fig. 2(a), this filter consists of an inner aluminum disc (70 mm in diameter, 1.6 mm thick), which is centered on a lead disc (220 mm in diameter, 0.1 mm thick). The upper and lower 10 mm of the lead disc have been trimmed away to increase the fluence at the superior and inferior regions of the field. To provide sufficient rigidity, the two metallic discs are held between two polymethyl methacrylate (PMMA) plates (0.8 mm thick). Portions of the PMMA plates have been removed to increase the fluence at the superior and inferior regions of the field. Fig. 2(b) is a photograph of the flattening filter mounted on the LINAC's accessory tray.

### C. Rotating platform

A custom‐built rotating platform with a circular standing area 60 cm in diameter was built in the departmental machine shop for this technique. The top surface of the rotating platform is 30 cm above floor level, and for shorter patients, a hard foam spacer can be added to increase the platform's height to 50 cm above the floor level. The distance from the isocenter to the floor is 130 cm, and therefore the distance from the platform's top surface to the level of the isocenter can be set at either 80 cm or 100 cm. The platform has a small 112‐V variable speed motor (TRW Electronics, Redondo Beach, CA) that is geared to deliver rotational rates up to 7 rpm. The speed is selected by varying the input current to the motor. A choice of 3 rpm is set so as to provide a compromise between the high speed required for the dosimetric independence of start and stop positions, and the low speed required for patient safety and comfort. To physically assist and support the patient, a grip bar is affixed to the wall to aid the patient during setup, and a suspended rotating handlebar is centered midair above the axis of rotation to aid patients in retaining their balance while standing on the rotating platform during the rotational treatment.

### D. Machine output

Given the extended SSD used in this technique, a higher output is required from the LINAC. The necessary output is possible using the optional 6 MeV HDTSe mode provided by the manufacturer. The HDTSe mode operates through a separate dose servo board that alters several LINAC operating parameters to produce a higher dose rate:

- First, the internal LINAC pulse‐forming network frequency is doubled to 360 Hz from 180 Hz to effectively double the number of accelerated electrons per unit time.
- Second, the electron gun filament current is increased to boost the number of electrons ejected from the gun per pulse. The value of the gun filament current is proprietary.
- Third, the number of dropped pulses in a typical cycle is reduced, thereby providing increased beam production efficiency.

Each of the foregoing changes increases the number of electrons produced without altering either the beam's shape or energy distribution, so that the HDTSe mode exhibits exactly the same percentage depth doses and off‐axis ratios at an SSD of 100 cm as the standard 6 MeV electron mode does.

Typically, in the standard photon and electron modes, the nominal output of the LINAC is adjusted to yield 1 cGy per monitor unit (MU) at the depth of dose maximum in water at 100 cm SSD. In the 6 MeV HDTSe mode, on the other hand, the LINAC yields 3.1cGy/MU at the depth of maximum dose at 100 cm SSD. Furthermore, in contrast with the conventional machine repetition rate of 400 MU/min, the HDTSe mode operates at 888 MU/min. Consequently, the nominal dose rate of the LINAC at SSD 100 cm for the 6 MeV HDTSe mode is approximately 2750 cGy/min as opposed to the conventional LINAC dose rate of 400 cGy/min for the conventional 6 MeV electron mode.

### E. Dose prescription

Patients are typically prescribed either 36 Gy or 40 Gy, in fractions of 2 Gy. They usually receive four rotational fractions per week for a total treatment duration of 5 weeks. Areas of the skin that are known to receive a lower‐than‐prescribed dose are supplemented with additional treatment fields using a conventional 6 MeV electron technique.^(8)^ The most frequently added electron boost fields include the vertex of the head, the palms of the hands, the soles of the feet, and the perineum. Other boost fields may include the axilla, infra‐mammary folds, or other similar locations that may be physically blocked from the rotational electron beam delivery.

## III. RESULTS

### A. Percent depth dose

**Figure 3:**
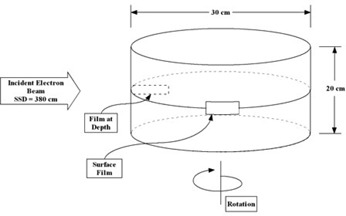
A schematic of the phantom used to measure rotational percent depth doses with strips of Gafchromic EBT (external beam therapy) radiochromic film. SSD=source‐to‐surface distance.

Stationary percent depth doses (PDDs) were measured with a PPC40 plane‐parallel chamber (Scanditronix–Wellhofer, Schwarzenbruck, Germany) embedded in Solid Water (Gammex RMI, Middleton, WI). Rotational PDDs were all measured with strips of Gafchromic EBT (external beam therapy) radiochromic film (International Specialty Products, Wayne, NJ) placed between two Solid Water cylinders (30 cm in diameter, 10 cm thick), as illustrated in Fig. 3. The exposed radiochromic film was scanned with an Arcus II (Agfa, Mortsel, Belgium) flatbed document scanner used in the transmission mode, and the electron dose was determined according to the protocol formulated by Devic et al.^(9)^ A polynomial calibration curve was obtained by irradiating a series of films to known doses of electrons ranging from 15 cGy to 600 cGy.

**Figure 4:**
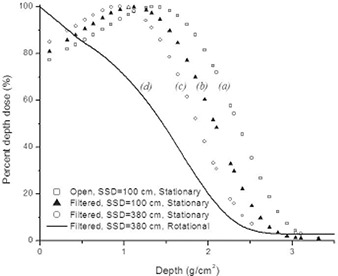
Percent depth dose (PDD) plots. (a) The stationary standard 6 MeV electron beam PDD measured at a source‐to‐surface distance (SSD) of 100 cm with a electron applicator and a 10 cm×10 cm field size. (b) The stationary high‐dose total skin electron (HDTSe) PDD measured with the flattening filter in place at an SSD of 100 cm with the photon collimator jaws set to 40 cm×40 cm. (c) The stationary HDTSe PDD measured with the flattening filter in place at an SSD of 380 cm with the photon collimator jaws set to 40 cm×40 cm. (d) The rotational HDTSe PDD measured with the flattening filter in place at an SSD of 380 cm with the photon collimator jaws set to 40 cm×40 cm.

Fig. 4 shows 6 MeV electron depth dose distributions measured under various conditions in water‐equivalent phantoms. Curves for stationary beams are represented with data points: (a) is a 10 cm×10 cm clinical beam produced with an electron cone measured at an SSD of 100 cm for the standard‐mode 6 MeV beam operating at 400 MU/min; (b) is the large‐field electron beam (no electron cone, but electron flattening filter in place) at an SSD of 100 cm with the photon collimator jaws set to 40 cm×40 cm; and (c) is the same conditions as (b), but measured at an SSD of 380 cm. The solid curve (d) in Fig. 4 is for a rotational beam measured with radiochromic film in a cylindrical phantom at an SSD of 380 cm with the photon collimator jaws set to 40 cm×40 cm. Curves (b), (c), and (d) were measured with the 6 MeV HDTSe electron mode with an output set to 888 MU/min.

As shown in Fig. 4, the conventional 6 MeV clinical electron beam under reference conditions has a $z_{\text{max}}$ of $1.3\;g/{\text{cm}}^{2}$, an $R_{50}$ of $2.3\;g/{\text{cm}}^{2}$, and an $R_{p}$ of $3.0\;g/{\text{cm}}^{2}$. With HDTSe at an SSD of 100 cm and the custom flattening filter in place, the PDD shows a $z_{\text{max}}$ of $1.1\;g/{\text{cm}}^{2}$, an $R_{50}$ of $2.1\;g/{\text{cm}}^{2}$, and an $R_{p}$ of $3.0\;g/{\text{cm}}^{2}$. At the extended SSD of 380 cm, the filtered beam shows a $z_{\text{max}}$ of $0.9\;g/{\text{cm}}^{2}$, an $R_{50}$ of $1.8\;g/{\text{cm}}^{2}$, and an $R_{p}$ of $2.8\;g/{\text{cm}}^{2}$. Lastly, the rotational PDD has a surface dose at 100%, an $R_{50}$ of $1.5\;g/{\text{cm}}^{2}$, and an $R_{p}$ of $2.3\;g/{\text{cm}}^{2}$. Of additional clinical importance in total skin treatments is the bremsstrahlung dose, because this dose represents the whole‐body dose burden that will be received by the patient. For our RTSEI technique, the highest bremsstrahlung dose is delivered at the central axis and is of the order of 2.7% at a depth of $5\;5/g^{\text{cm}}$. Table 1 summarizes the data for $z_{\text{max}}$, $R_{50}$, $R_{p}$, and bremsstrahlung dose.

**Table 1:** A summary of dosimetric properties measured in Solid Water^a^ for four beam arrangements

*6 MeV Beam SSD (cm)*	*(1) Stationary 100*	*(2) Stationary 100*	*(3) Stationary 380*	*(4) Rotational 380*
Field size and parameters	Electron cone 10 cm×10 cm No flattening filter	No electron cone Jaws at 40 cm×40 cm With flattening filter	No electron cone Jaws at 40 cm×40 cm With flattening filter	No electron cone Jaws at 40 cm×40 cm With flattening filter
Clinical beam	Yes, standard RT	No	Yes, TSEI	Yes, RTSEI
Machine mode	6 MeV Standard	HDTSe	HDTSe	HDTSe
$z_{\text{max}}({\text{cm}}^{2}/g)$	1.3	1.1	0.9	0
$R_{50}({\text{cm}}^{2}/g)$	2.3	2.1	1.8	1.5
$R_{p}({\text{cm}}^{2}/g)$	3.0	3.0	2.8	2.3
${\text{PDD}}_{\text{brems}}(z=5\;g/{\text{cm}}^{2})$	1.0	1.1	3.0	2.7

a Gammex RMI, Middleton, WI.

SSD=source‐to‐surface distance; RT=radiation therapy; TSEI=total skin electron irradiation; RTSEI=rotational total skin electron irradiation; HDTSe=high‐dose total skin electron mode; ${\text{PDD}}_{\text{brems}}=\text{percent}$ depth dose bremsstrahlung.

### B. Off‐axis ratios

**Figure 5:**
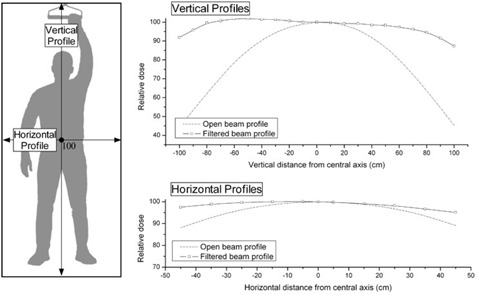
Off‐axis ratios showing the effect of the flattening filter in both the horizontal and vertical directions. The measurements were all performed at $z_{\text{max}}$ in Solid Water (Gammex RMI, Middleton, WI) and at a source‐to‐surface distance of 380 cm.

**Figure 6:**
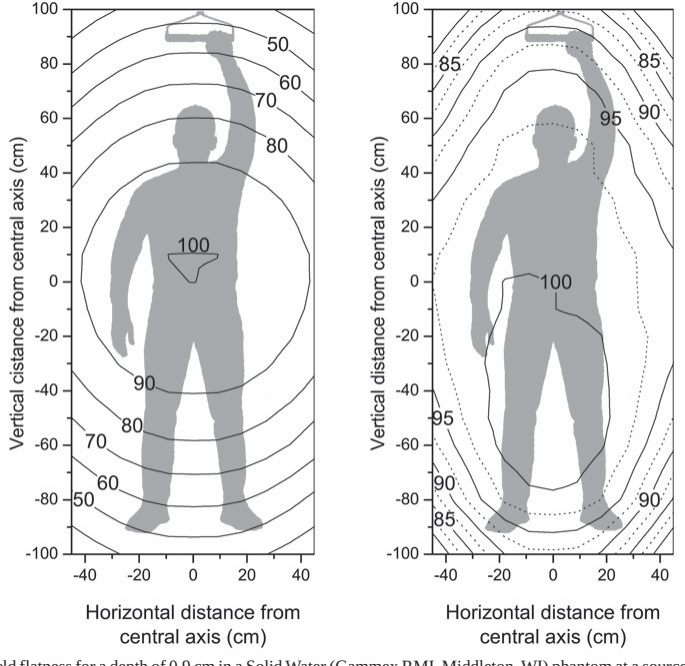
Field flatness for a depth of 0.9 cm in a Solid Water (Gammex RMI, Middleton, WI) phantom at a source‐to‐surface distance of 380 cm for (a) the unfiltered open beam, and (b) the flattened beam used clinically.

Dose profiles and off‐axis ratios for the stationary large‐field electron beam were measured with a PPC40 ionization chamber at a depth of 0.9 cm in a Solid Water phantom at an SSD of 380 cm. An array of measurements, each with 5‐cm separation, are plotted for the open field and for the flattened field in Figs. 4 and 5. Fig. 4 shows our measured profiles for the vertical and horizontal central beam axes: dashed curves represent the open (unfiltered) electron beam and solid curves represent the electron beam flattened with our custom‐made electron flattening filter (Fig. 2). The field flatness for the open field and the filtered field can be seen by the isodose contour plots in Fig. 6(a) for the unflattened beam and in Fig. 6(b) for the flattened beam used clinically. In the case of the modified beam, we see that a typical patient would be entirely covered by the 90% level and nearly all but forearms and feet would be irradiated by the 95% level.

### C. Dose rate versus SSD

During a patient's treatment, relatively small variations in SSD will occur because of the inherent motion of the patient. For this reason, a brief investigation into the change in dose rate versus SSD should be considered. The output of the 6 MeV HDTSe beam measured at a depth of $z_{\text{max}}$ can be expressed as a continuously decreasing function with respect to distance from the nominal isocenter of the LINAC. When fit with an exponential function, the output versus SSD varies as a power of 2.2 over the range of clinical interest from SSD 330 cm to SSD 430 cm, such that, when 330 cm ≤SSD ≤430 cm,

(1) $$D=D_{0}{\left(\frac{SSD_{0}}{SSD}\right)}^{2.2},$$

where *D* is the dose at the SSD of interest, and $D_{0}$ is the dose at the nominal ${\text{SSD}}_{0}$ of 380 cm.

### D. Rotational factor and beam calibration

The absolute output for the HDTSe 6 MeV stationary electron beam using the custom flattening filter was determined with a parallel‐plate ionization chamber that was cross‐calibrated in a high‐energy stationary electron beam at an SSD of 100 cm according to the American Association of Physicists in Medicine TG‐51 protocol to determine the product $\left(k_{\text{ecal}}N_{D,w}^{\text{Co}-60}\right)$. The stationary extended SSD HDTSe electron beam was then calibrated in terms of the reference absorbed dose to water in centigrays per 1000 MU using the TG‐51 protocol parameters,^(10)^ as expressed in equation 2:

(2) $$D_{\text{water}}=M_{\text{TP}}\times P_{\text{pol}}\times P_{\text{ion}}\times k_{R_{50}}^{\prime }\times (k_{\text{ecal}}N_{D,W}^{\text{Co}-60}).$$

The stationary beam calibration was carried out for our RTSEI beam delivered with the gantry at 270 degrees, the collimator set at 40 cm×40 cm and rotated to 45 degrees, the couch at 45 degrees, the custom flattening filter in place, and a plane‐parallel chamber embedded in Solid Water at the depth of maximum dose (9 mm). In this manner, the absorbed dose delivered per 1000 MU on the beam's central axis at an SSD of 380 cm was determined to be 54.0 cGy.

To find the dose rate to a point of skin on the patient undergoing rotation, we multiply the stationary dose rate by a factor. Intuitively, because any point on the patient is directly in the radiation field for one half of the period of rotation and is eclipsed by the other parts of the body for the other half of the rotation, we expect this factor to be near 0.5.

This factor was recently measured using two dosimetry systems: Gafchromic EBT film and OneDose MOSFET (metal oxide semiconductor field effect transistor) detectors (Sicel Technologies, Morrisville, NC). In each measurement, this rotational factor was determined to be the ratio of the dose rate at depth of dose maximum in Solid Water for a stationary electron beam and the dose rate recorded at the surface of a Solid Water cylinder 29.7 cm in diameter for a rotational electron beam. Each of these dose rates was measured with the gantry at 270 degrees, the collimator set at 40 cm×40 cm and rotated to 45 degrees, the couch at 45 degrees, and the custom flattening filter in place. The ratio determined from the Gafchromic EBT film was 0.443, and the ratio determined from the OneDose MOSFET detectors was 0.448. These two independent measurements confirm the previously‐determined ratio of 0.45,^(1)^ measured with radiographic film and thermoluminescent dosimeters for our original RTSEI technique.

Multiplication of the stationary output of 54.0 cGy/1000 MU by this rotational factor of 0.45 results in a rotational dose rate of 24.3 cGy/1000 MU at the surface of the Solid Water cylinder 30 cm in diameter. At the HDTSe dose rate of 888 MU/min, the resulting treatment time is 9.5 minutes for a typical treatment fraction prescription of 200 cGy delivered with 8230 MU.

Because the custom flattening filter is mounted downstream from the LINAC's transmission ionization chamber, there is a risk that treatment might be set up incorrectly without the filter in place, or that the filter might break and become displaced during treatment, resulting in a higher‐than‐expected and non‐uniform dose. As a precaution against this unlikely event, a secondary dosimetry monitoring system consisting of an independent NE 2571 Farmer type chamber (Nuclear Enterprises, Edinburgh, Scotland) and a Model 530 electrometer (Victoreen, Cleveland, OH) has been installed. The chamber is mounted on a support system in the treatment beam slightly lateral to the patient near the nominal treatment SSD of 380 cm. During beam calibration, a relative factor is determined for this particular setup with the secondary dosimetry system, and in this manner, day‐to‐day treatments can be monitored for consistency and to ensure increased safety. This system also permits a set of checks to be performed before patient treatment so as to ensure that the technique is ready for proper clinical beam delivery.

### E. Verification on a Rando anthropomorphic phantom

Table 2 shows results of point‐dose surface measurements at various positions on a humanoid phantom, performed both with small Gafchromic EBT film squares and OneDose MOSFET detectors. Data are expressed as a percent value relative to the expected prescription dose of 200 cGy at the anterior abdominal position at the level of the umbilicus. With the exception of a point in the anterior neck region, dose delivery within the criteria of ±10% is achieved. The anterior neck receives a higher dose because of its inherent geometry, which presents a thinner cross‐section, resulting in a higher oblique beam incidence and a subsequently higher dose.

**Table 2:** Surface point‐dose measurements on Rando phantom,^a^ recorded at a source‐to‐surface distance of 380 cm using the 6 MeV high‐dose total skin electron (HDTSe) mode, and expressed as a percentage of the 200 cGy prescription (Rx) dose

*Site*	*Position*	*Film dose (% of Rx dose)*	*MOSFET dose (% of Rx dose)*
Abdomen	Anterior	102	102
	Posterior	97	97
	Left	108	104
	Right	104	100
Neck	Anterior	118	111
	Posterior	99	100
	Left	105	107
	Right	110	102
Head	Anterior	101	106
	Posterior	99	102
	Left	97	102
	Right	96	96

a The Phantom Laboratory, Salem, NY.

MOSFET=metal oxide semiconductor field effect transistor.

**Figure 7:**
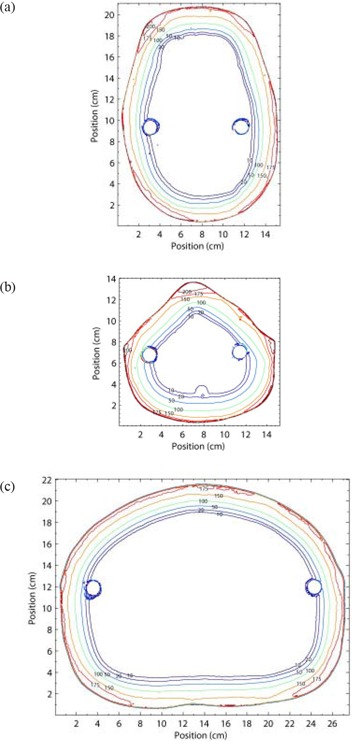
Isodose plots of the head, neck, and abdomen dose distributions measured in the Rando phantom (The Phantom Laboratory, Salem, NY) at a source‐to‐surface distance of 380 cm using the 6 MeV high‐dose total skin electron (HDTSe) mode with the rotating platform and the custom flattening filter to deliver a 200 cGy prescription dose. Lines represent the absolute dose values in centigrays measured using Gafchromic EBT (external beam therapy) film. The circular artifacts 1 cm in diameter are a result of vertical channels in the phantom through which support rods were positioned.

Cross‐sectional isodose distributions of the head, neck, and abdomen have been measured for the full rotation technique and are shown in Fig. 7. The absolute dose readings obtained when a 200 cGy prescription dose is delivered under RTSEI conditions are shown.

## IV. DISCUSSION

The optional commercially available HDTSe mode operating at a nominal energy of 6 MeV provides an adequate dose rate for the RTSEI treatment at extended SSD. The technique becomes clinically acceptable when a custom‐built flattening filter is used. The typical prescription dose of 200 cGy is delivered in 9.5 minutes at an SSD of 380 cm with the custom flattening filter in place. Given a patient rotation speed of 3 rpm, the patient undergoes approximately 28 full revolutions per treatment. The patient's rotation commences before the beam is turned on and continues beyond the end of the dose delivery. There is no mechanism for coordinating either the beam‐on time, or the delivery rate, between the LINAC and the rotating platform. However, over the 18 or so fractions, the start and stop positions will vary sufficiently to smooth out and reduce this effect, and the dose inhomogeneity resulting from any asymmetry between the start and stop angles should be negligible and are thus ignored in our clinical use of the RTSEI technique.

Before treatment, patients are dressed in a disposable body gown made of thin paper. The dosimetric effect of this gown has been determined to be negligible, yet it provides the patient some measure of comfort and dignity during treatment. According to the prescription, internal tungsten eye shields (Radiation Products Design, Albertville, MN) and lead shields 1 mm thick for the fingernails and toenails are affixed for the desired number of treatment fractions. In addition, a lead vertex shield 5 mm thick is affixed to the top of the skull to provide a defined region for the vertex boost for every treatment fraction. Without this vertex shield, boosting of the vertex region would be difficult, because the tangential nature of the beam in this region makes for a large dose gradient.

Once the shields are in place, the patient is assisted onto the platform, where 1 of 3 sets of paired foot positions on the platform is chosen. In addition, either the right or left hand is used to grasp the overhead support. In this manner, the combination of 2 hand positions and 3 foot positions gives a total of 6 body positions that are used on sequential days for treatment during the course of the 18 or so treatments. The purpose of these 6 body positions is to reduce the effect of self shielding by the arms and legs on the abdomen and inner thighs respectively.^(8)^ ^,^ ^(11)^ Patients are typically given RTSEI treatments on Monday, Tuesday, Thursday, and Friday. Boosts to the vertex, soles and palms, perineum, infra‐mammary folds, and elsewhere are delivered as required throughout the 5 weekdays. These boosts are delivered with a stationary setup at an SSD of 100 cm with an electron applicator in place using the standard 6 MeV (non‐HDTSe) electron beam.

Compared with our former technique, which required modifications to the LINAC's hardware, the use of Varian's HDTSe mode is very practical in our clinical environment. We are able to treat the RTSEI patients in the normal clinical day, with minimal setup time for the apparatus, and treatments may be delivered using our record‐and‐verify system (VARIS: Varian Medical Systems). We generally deliver fewer than 200 RTSEI treatment fractions in a year, and this optional high‐dose rate mode has so far proved to be a reliable and robust treatment modality.

## V. CONCLUSIONS

We recently transferred our RTSEI technique from a Clinac 18 LINAC to a newer Clinac 21EX LINAC equipped with a commercially‐available HDTSe mode. The dosimetric properties of the current technique closely mimic the ones from the originally‐used technique. The stationary beam absolute calibration in water has been determined to be 54.0 cGy/1000 MU at the extended SSD of 380 cm, and we have determined the rotational dose rate to be 24.3 cGy/1000 MU at the patient's surface. Because the LINAC operates at 888 MU/min, treatments are delivered over approximately 9.5 minutes. For the 6 MeV HDTSe mode at an SSD of 380 cm, the stationary electron beam yields a $z_{\text{max}}$ of $0.9\;g/{\text{cm}}^{2}$, an $R_{50}$ of $1.8\;g/{\text{cm}}^{2}$, and an $R_{p}$ of $2.8\;g/{\text{cm}}^{2}$. The rotational electron beam yields a surface dose at 100%, an $R_{50}$ of $1.5\;g/{\text{cm}}^{2}$, and an $R_{p}$ of $2.3\;g/{\text{cm}}^{2}$.

The bremsstrahlung dose at a depth of $5\;g/{\text{cm}}^{2}$ for the rotational treatment is 2.7% of the maximum dose. Since we started to use the commercially available 6 MeV HDTSe mode for our RTSEI technique on the Varian 21EX in 2005, we have treated 15 patients. To date, we have treated 170 patients with the RTSEI technique: 155 with the original Clinac‐18 LINAC and 15 with the updated technique carried out on the modern Clinac‐21EX LINAC.
